# Caveolin-1 regulates the ASMase/ceramide-mediated radiation response of endothelial cells in the context of tumor–stroma interactions

**DOI:** 10.1038/s41419-020-2418-z

**Published:** 2020-04-09

**Authors:** Julia Ketteler, Alina Wittka, Daniela Leonetti, Victoria Veas Roy, Hala Estephan, Patrick Maier, Henning Reis, Carsten Herskind, Verena Jendrossek, Francois Paris, Diana Klein

**Affiliations:** 1 Institute of Cell Biology (Cancer Research), University of Duisburg-Essen, University Hospital, Virchowstrasse 173, 45122 Essen, Germany; 2 CRCINA, INSERM, CNRS, Université de Nantes, Nantes, France; 3 Department of Radiation Oncology, University Medical Center Mannheim, Medical Faculty Mannheim, Heidelberg University, Theodor-Kutzer-Ufer 1-3, 68167 Mannheim, Germany; 4 Institute of Pathology, University of Duisburg-Essen, University Hospital, Hufelandstr. 55, 45122 Essen, Germany

**Keywords:** Cancer therapeutic resistance, Apoptosis, Cancer microenvironment, Translational research

## Abstract

The integral membrane protein caveolin-1 (CAV1) plays a central role in radioresistance-mediating tumor–stroma interactions of advanced prostate cancer (PCa). Among the tumor–stroma, endothelial cells (EC) evolved as critical determinants of the radiation response. CAV1 deficiency in angiogenic EC was already shown to account for increased apoptosis rates of irradiated EC. This study explores the potential impact of differential CAV1 levels in EC on the acid sphingomyelinase (ASMase)/ceramide pathway as a key player in the regulation of EC apoptosis upon irradiation and cancer cell radioresistance. Enhanced apoptosis sensitivity of CAV1-deficient EC was associated with increased ASMase activity, ceramide generation, formation of large lipid platforms, and finally an altered p38 mitogen-activated protein kinase (MAPK)/heat-shock protein 27 (HSP27)/AKT (protein kinase B, PKB) signaling. CAV1-deficient EC increased the growth delay of LNCaP and PC3 PCa cells upon radiation treatment in direct 3D spheroid co-cultures. Exogenous C6 and C16 ceramide treatment in parallel increased the growth delay of PCa spheroids and induced PCa cell apoptosis. Analysis of the respective ceramide species in PCa cells with increased CAV1 levels like those typically found in radio-resistant advanced prostate tumors further revealed an upregulation of unsaturated C24:1 ceramide that might scavenge the effects of EC-derived apoptosis-inducing C16 ceramide. Higher ASMase as well as ceramide levels could be confirmed by immunohistochemistry in human advanced prostate cancer specimen bearing characteristic CAV1 tumor–stroma alterations. Conclusively, CAV1 critically regulates the generation of ceramide-dependent (re-)organization of the plasma membrane that in turn affects the radiation response of EC and adjacent PCa cells. Understanding the CAV1-dependent crosstalk between tumor cells and the host-derived tumor microvasculature and its impact on radiosensitivity may allow to define a rational strategy for overcoming tumor radiation resistance improving clinical outcomes by targeting CAV1.

## Introduction

The mode of organization within the plasma membrane is essential for its role within several cellular processes. Cholesterol, as a major plasma membrane component, critically affects the plasma membrane organization^1^. Intermolecular interactions between sphingolipids and cholesterol molecules result in the lateral and local association of these lipids, finally forming sphingolipid- and cholesterol-enriched membrane domains termed lipid rafts^2,3^. Caveolae, non-planar specialized membrane microdomains (vesicular invaginations), form a subgroup of these lipid rafts. Herein, the integral membrane protein caveolin-1 (CAV1) was identified as a major structural component of caveolae and as cholesterol-binding protein CAV1 that stabilized those lipid rafts^4,5^.

Disturbance of the membrane organization, damage in membrane integrity, or lipid modifications have profound effects on lipid organization, membrane dynamics, and signaling. Cellular stress stimuli, and in particular ionizing radiation (IR), were shown to affect plasma membrane organization and especially raft microdomains^6–8^. Radiation generally increased membrane ceramide rapidly by directly activating acid sphingomyelinase (ASMase) that hydrolyzes sphingomyelin to ceramide in rafts^7^. An increased ceramide content then provokes extensive spatial lipid reorganization, resulting from the fusion of membrane lipid rafts into large ceramide-enriched domains^9^. In contrast to the sphingolipid- and cholesterol-enriched lipid rafts that mainly were involved in cell proliferation, the larger and more long-lived ceramide-enriched lipid rafts were predominantly involved in apoptosis^10–12^.

Resistance to apoptosis remains one major obstacle in cancer treatment. Herein, the tumor microenvironment is increasingly recognized not just as a growth-supporting tumor compartment but as a key determinant of radiation therapy (RT) resistance^13–16^. Among the cell types within the tumor–stroma, ECs were shown to be critical determinants of the radiation response of tumors^9,17–20^. IR exposure induced acute vascular damage and EC apoptosis, leading to severe EC loss finally resulting in vascular dysfunction^17,20–22^. Mechanistically, the p38 MAPK signaling pathway is essential for the EC response to stress stimuli including IR^23^. RT-induced p38 activation in EC even reduced the survival protein AKT/PKB (protein kinase B), thereby promoting pro-apoptotic signaling^23,24^. RT-induced p38 pathway-dependent EC apoptosis was further linked to ASMase/ceramide signaling and plasma membrane reorganization^25^. ECs were characterized by high levels of ASMase expression that may account for the increased vulnerability of the endothelium to radiation-induced apoptosis^26,27^.

CAV1 gained increasing attention in cancer therapy because CAV1 expression levels strongly increase in malignant epithelial cells of many solid tumors at advanced tumor stages^28–30^. In parallel, a loss of stromal CAV1 could be observed, which correlated with tumor progression and therapy resistance, especially in prostate cancer (PCa), and may therefore be suited as a prognostic marker^29,31–33^. However, the vascular compartment was characterized by a stable expression of CAV1^19^. Our own previous work revealed an association between CAV1 in EC and the RT response: silencing of CAV1 increased the radiation-induced EC death^19^. CAV1-deficient EC and the respective blood vessels were further characterized by a less stabilized, pro-angiogenic phenotype that facilitated tumor growth^19,34,35^. Even more important, these CAV1-deficient tumor ECs were more sensitive to RT and promoted an improved tumor RT response^19^. Lowering CAV1 levels in tumor EC may thus be suited to improve the outcome of RT in cancer.

Here, we explored a potential link between the CAV1-dependent radiation response of EC and signaling mediated by ceramide-enriched platforms that in turn lead to the activation of downstream -signaling molecules launching the apoptotic process. Furthermore, we investigated how those CAV1-dependent signaling pathways in EC affect the radiation response of adjacent PCa cells.

## Results

### A differential CAV1 expression in EC impacts on the AKT/PKB cell survival and the apoptosis-regulating p38 pathway upon radiation

**Fig. 1:**
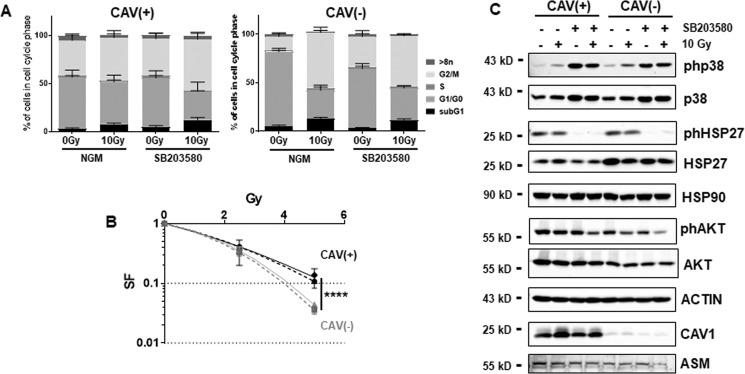
Apoptosis-prone CAV1-deficient EC expresses higher levels of HSP27 while the survival protein AKT/PKB is downregulated. CAV1-proficient [CAV1(+)] and CAV1-deficient [CAV1(−)] ECs were subjected to radiation treatment (0 Gy control or 10 Gy) with or without p38 inhibitor treatment (SB203580, 10 µM). **a** Cell cycle phases and apoptotic cells (sub-G1) were analyzed by flow cytometry 48 h after irradiation. Graphs consist of data from 3 to 4 individual experiments (with SEM). *p < 0.05 (for G1/G0 and G2/M: 0 Gy vs. 10 Gy CAV(+) SB203580) and ****p < 0.001 (for G1/G0 and G2/M: 0 Gy vs. 10 Gy CAV1(−) NGM and SB203580) by two-way ANOVA with post hoc Tukey multiple-comparison test (not depicted). **b** ECs were plated for colony formation assay, pretreated for 2 h with SB203580, irradiated with indicated doses, and subsequently further incubated for an additional 10 days. Data show the surviving fractions (SF) from three independent experiments (means ± SD). Dashed lines depict the survival fraction upon p38 inhibition. ****p < 0.001 by two-way ANOVA with post hoc Tukey multiple-comparison test. **c** Whole-cell lysates were used for Western blot analysis of the p38/MAPK and AKT/PKB pathways by detecting the indicated proteins in control and 10 Gy irradiated cells 48 h after treatment. In addition, CAV1 expression levels were measured at the same time points. β-ACTIN was used as a loading control. Representative blots of 3–4 individual experiments are shown.

ECs with a reduced CAV1 expression were more sensitive to IR as previously reported^19^ and as again confirmed here using the EC line AS-M5 without or with shRNA knockdown of CAV1 (Fig. 1a, b). To further understand a potential cross-regulation of the well-known EC stress response p38 pathway and cell survival pathways, we pretreated the EC with a p38 inhibitor (SB203580) 1–2 h prior radiation treatment. The respective cell cycle analyses 48 h after radiation revealed that radiation reduced the number of CAV(+) EC in G1/G0 only minimally, while cells in the G2/M phase increase (Fig. 1a). Upon p38 inhibitor treatment, these alterations became significant. Radiation significantly reduced the CAV1(−) cell numbers in G1/G0 and increased CAV1(−) EC in G2/M, which was not further affected upon p38 inhibition. The reduced Cav1 levels resulted in increased apoptosis rates and reduced survival fractions (Fig. 1a, b). CAV1 dependency of the p38 cell death and the AKT/PKB survival pathway was investigated by Western blot analysis (Fig. 1c). Forty-eight hours after IR, increased p38 phosphorylation levels were detected in both CAV1 EC variants although the total p38 levels were not affected. The p38 downstream effector HSP27 was already phosphorylated in control conditions in both EC variants and IR causing no further increase. Apoptosis-prone CAV1-deficient EC, however, expressed higher levels of HSP27. p38 inhibitor treatment efficiently limited HSP27 phosphorylation. Of note, phosphorylation of AKT as well as total AKT protein levels were reduced in CAV1-deficient EC. A decreased expression of phosphorylated AKT was further prominent 48 h after IR, the time point of radiation-dependent apoptosis execution. A further (although slight) reduction was seen in both CAV1 EC variants upon p38 inhibitor treatment in combination with radiation. HSP90 however, that was shown to coordinate the trafficking and regulation of diverse signaling proteins in EC, was not affected by a differential CAV1 content of EC or upon radiation. In summary, radiation caused an activation of p38 MAPK. Apoptosis-prone CAV1-deficient EC was characterized by increased HSP27 levels and decreased AKT levels that might already indicate that HSP27 and AKT levels as well as their interactions are important for the regulation of cell survival versus cell death.

We then used a siRNA approach to knock down HSP27 in both EC variants (Supplemental Fig. S1). Although HSP27 siRNA treatment efficiently reduced total HSP27 levels, no additional cell cycle and cell death alterations were detected upon IR. Again, an increased p38 activity was detected in both EC variants as shown by increased p38 phosphorylation upon irradiation that were not affected by silencing HSP27. No additional effects on AKT were detected upon HSP27 silencing and IR, which suggests that HSP27 acts downstream of AKT.

### Endothelial CAV1 deficiency increased the RT-induced activation of ASMase leading to increased ceramide generation

**Fig. 2:**
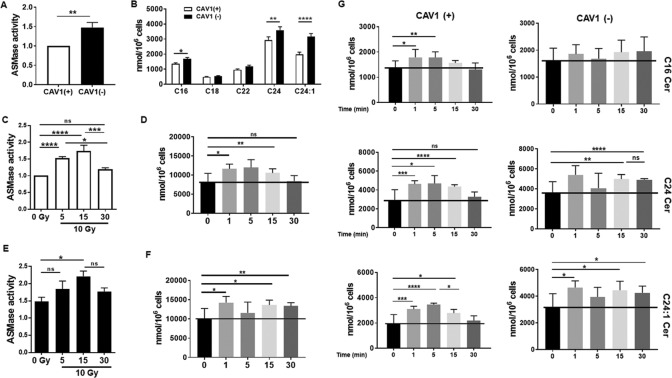
ASMase activity and ceramide generation are increased in CAV1(−) EC and increase in CAV1(+) EC in a time-dependent manner after IR. **a** ASMase enzymatic activity was measured in AS-M5 CAV1(+) and CAV1(−) cells in control conditions (*n* = 13). CAV1(−) activity is shown in relation to CAV1(+) cells, that was set at 1. **p < 0.01 by paired *t* test (two-tailed). **b** Overview of the most prominent detected ceramide species by LC–MS in CAV1(+) and CAV1(−) EC (*n* = 3, SEM). *p < 0.05, **p < 0.01, and ****p < 0.001 by two-way ANOVA with post hoc Tukey multiple-comparison test. **c**, **e** ASMase enzymatic activity was analyzed after 10-Gy IR. ASMase activity of CAV1(+) (**c**) and CAV1(−) (**e**) EC is shown in relation to CAV1(+) unirradiated samples (set as 1) 5, 15, and 30 min after IR treatment (*n* = 3–4). *p < 0.05, ***p < 0.005, and ****p < 0.001 by one-way ANOVA with post hoc Tukey multiple-comparison test; ns not significant. **d**, **f** Timeline of the total ceramide levels generated by CAV1(+) (**d**) and CAV1(−) (**f**) EC after IR treatment. Samples were taken 1, 5, 15, and 30 min after 10-Gy irradiation (*n* = 3, SD). All control (0-Gy) samples were pooled (*n* = 12). Statistical analysis was done by using Student’s *t* test with Welch’s correction (*p < 0.05, **p < 0.01). **g** Timeline of ceramide species C16, C24, C24:1, and total ceramide levels generated by CAV1(+) and CAV1(−) EC after 10-Gy irradiation (*n* = 3, SD). Samples were taken at indicated time points. Statistical analysis was done by using Student’s *t* test with Welch’s correction (*p < 0.05, **p < 0.01, ***p < 0.001, ****p < 0.001).

EC apoptosis was shown to depend on the early generation of ceramide^25,27,36^. Moreover, ECs with reduced CAV1 expression levels were more apoptosis-prone and thus more sensitive to IR^19^. Therefore, we first investigated the activity of ASMase and subsequent ceramide generation upon IR treatment in the EC with differential CAV1 levels (Fig. 2). Although total ASMase protein levels showed similar amounts in CAV1-proficient and CAV1-deficient EC (Fig. 1c), a significantly increased activity of the enzyme was detected in CAV1(−) EC (Fig. 2a). The resulting generation of different ceramide species was quantified and analyzed using liquid chromatography–mass spectrometry (LC–MS/MS) (Fig. 2b and Supplemental Fig. S2). ECs have been described to abundantly express the ceramide species C16, C24, and C24:1, which was confirmed in both EC CAV1 variants (Fig. 2b). Levels of C16, C24, and C24:1 ceramide were significantly increased in CAV1-deficient EC. In response to radiation, CAV1(+) EC showed a time-dependent significant increase in ASMase activity with a peak in activity at 15 min after IR. In total, 30 min after IR, these levels were decreasing nearly to control levels (Fig. 2c). In CAV1(−) EC, the already-increased ASMase activity gained a further increase upon IR (with a peak at 15 min upon IR) and the increased ASMase activity was maintained over time (Fig. 2e). The resulting ceramide levels were increased accordingly (Fig. 2d, f). Following IR, a rapid accumulation of ceramide was observed (Fig. 2d, f, g). At early time points after IR (1–15 min), the total ceramide levels as well as levels of the prominent species C16, C24, and C24:1 were promptly enhanced. Herein, ceramide generation reached a maximum of 5 min after treatment. In CAV1(+) EC, these increased ceramide levels were reduced to control levels 30 min after IR (Fig. 2d, g). In contrast, in CAV1(−) cells, ceramide generation was stabilized at the later time points (Fig. 2f, g). Conclusively, a reduction of CAV1 in EC increased ASMase activity, finally leading to increased ceramide generation. Therefore, we hypothesized that CAV1 deficiency leads to a defective ceramide homeostasis with an increase of pro-apoptotic ceramide signaling upon stress induction by IR. Apart from radiation-induced sphingomyelin hydrolysis by ASMase, ceramides can be synthesized ceramide synthases (CerS). Each CerS is known to produce a subset of ceramides that differ in their fatty acyl chain length, and in turn have distinct roles in inducing cell death versus survival. We investigated the expressions of the six mammalian CerS in CAV1(+) and CAV1(−) EC at steady state and 48 h after radiation (Supplemental Fig. S2c). While CerS5 and CerS6, which account for ceramides with fatty acyl chain length up to C16, were expressed in both cell types at similar levels and further induced following radiation treatment, CAV1(+) EC showed increased basal expression levels of CerS2 and CerS4 (accounting for very long ceramides up to C22–C24).

Thus, differential CAV1 levels in EC came along with differential ceramide species. Most importantly, the CAV1-dependent induction of ASMase-mediated sphingomyelin hydrolysis to ceramide in response to IR resulted in elevated C16 ceramide levels together with increased C24 levels in CAV1(−) EC (ratios are depicted in Supplemental Fig. S2b), that in turn might account for increased radiosensitivity of those cells.

### CAV1 deficiency enhanced the formation of membrane lipid raft domains and associated p38/HSP27 signaling upon IR

**Fig. 3:**
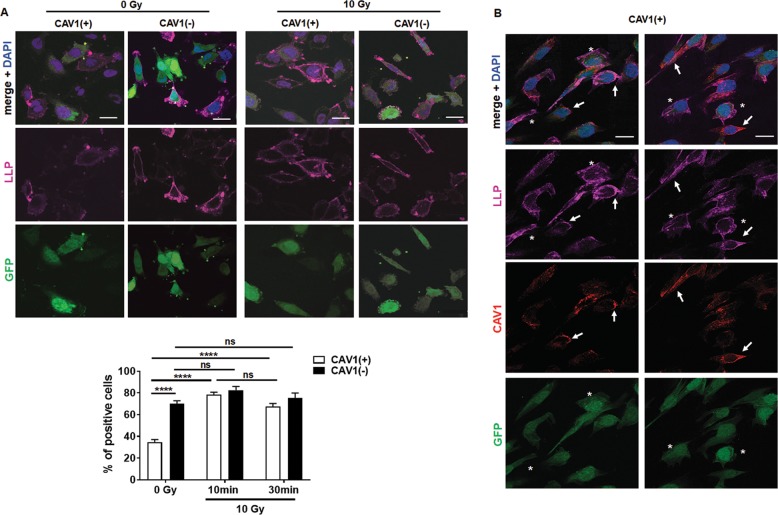
Large lipid platform (LLP) formation is elevated in CAV1(−) EC cells independently of stress induction. **a** Large lipid platform formation in CAV1(+) and CAV1(−) EC was visualized with Cholera-toxin B-subunit (purple) that binds ganglioside GM1 after irradiation with 0 (control) or 10 Gy. The lentiviral-transduced EC expresses GFP (green) as a reporter gene and nuclei were stained with DAPI (blue). Representative images (10 min after irradiation) of three individual experiments are shown (photographs). Quantification of LLP formation was done by counting positive cells of control-irradiated (0 Gy) and irradiated (10 Gy) cells. The graph shows LLP formation 10 and 30 min after IR. In total, at least 50 cells/condition were quantified (*n* = 3, SEM). Statistical analysis was performed with two-way ANOVA followed by post hoc Tukey’s test (****p < 0.001). **b** In addition, co-staining of LLP (purple) and CAV1 (red) of GPF-labeled (green) CAV(+) EC upon 10-Gy irradiation was performed. Arrows point to CAV1-immunoreactive structures, whereas asterisks mark EC with lower CAV1 immunoreactivity. Representative images are shown. Nuclei were stained with DAPI (blue). Scale bar: 10 µm.

Next, we investigated ceramide-induced plasma membrane remodeling from discrete structures to large lipid platforms (LLP) by immunofluorescence staining of glycosphingolipid GM1 with a fluorescent-tagged cholera-toxin-β-subunit (Fig. 3). Ten minutes after IR, agglutination and thus polarized GM1 was observed in CAV1(+) EC cells (Fig. 3a). An augmented GM1 clustering and thus the presence of LLP was observed in CAV1-deficient EC either with or without IR (Fig. 3a). Around 70% of CAV1(−) EC already contained LLP without IR, which was approximately the number of platforms/signalosomes CAV1(+) EC reached 10–30 min after IR (Fig. 3a). In CAV1-proficient EC, CAV1 was partially localized to those LLP (Fig. 3b, arrows). Of note, in CAV1(+) EC with a lower CAV1 immunoreactivity (Fig. 3b, asterisks) an increased GM1 clustering and so the presence of LLP was detected as seen in CAV1-deficient EC. The ceramide-induced LLP was further analyzed concerning a respective p38- and AKT-dependent downstream signaling (Supplemental Fig. S3). As shown for the 48-h time point after IR, p38 signaling in EC was not significantly affected by differential CAV1 levels but induced shortly after IR (5–30 min) (Supplemental Fig. S3a). Irradiated CAV1(−) EC was characterized by increased HSP27 levels. A reduction of phosphorylated AKT was already prominent 30 min after IR with overall reduced AKT levels in CAV1(−) EC, and a further slight reduction was seen in both variants upon p38 inhibitor treatment in combination with IR (Supplemental Fig. S3a). No influence of CAV1 on DNA damage response in EC upon IR could be observed (Supplemental Fig. S3b).

In summary, the results obtained up to now showed that the increased ceramide levels in EC cells, either based on RT-induced ASMase activity in CAV1-proficient EC or based on constitutively increased ASMase activity in CAV1-deficient EC, resulted in the formation of LLP, which then induced membrane remodeling and the downstream signaling. We speculated that in the normal steady-state situation of the plasma membrane, CAV1 inhibits the ceramide-induced LLP formation.

### CAV1-dependent ceramide generation in EC was paralleled by an increased radiation sensitivity of PCa

**Fig. 4:**
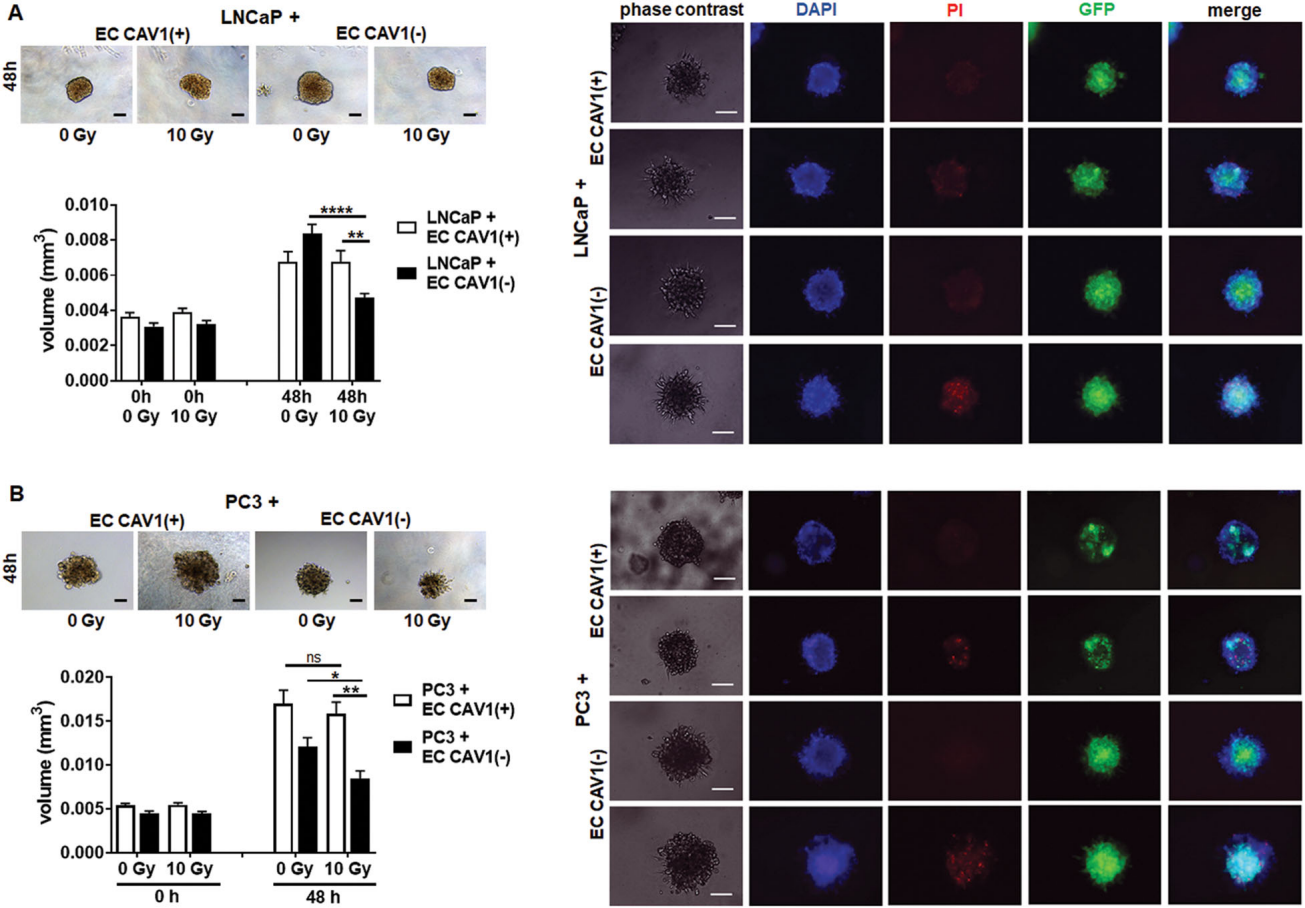
Direct interaction of Cav1-deficient EC with PCa cells increased RT-induced growth retardation. **a** LNCaP and **b** PC3 cells were co-cultured with CAV1(+) or CAV1(−) EC in hanging drops for 24 h. After formation of spheroids, cells were plated in growth factor-reduced Matrigel mixed with normal growth medium (1:2, v/v) and irradiated at 10 Gy. Pictures were taken at the time of irradiation (0 h) and 48 h later. Scale bar represents 50 µm. Representative phase-contrast images from three individual experiments are shown. Spheroid growth was measured and the respective volumes were calculated. Graphs depict the measurements from three independent experiments (*n* = 3, SEM) where at least ten spheroids per condition each were measured. *p < 0.05, **p < 0.01, ****p < 0.001 by two-way ANOVA followed by post hoc Tukey’s test. Cell death was analyzed afterward by fluorescence microscopy using propidium iodide. DAPI was used for nuclei staining. Representative phase-contrast images and simultaneously recorded fluorescent photographs from three individual experiments are shown (48 h’ time point). Scale bar represents 25 µm.

**Fig. 5:**
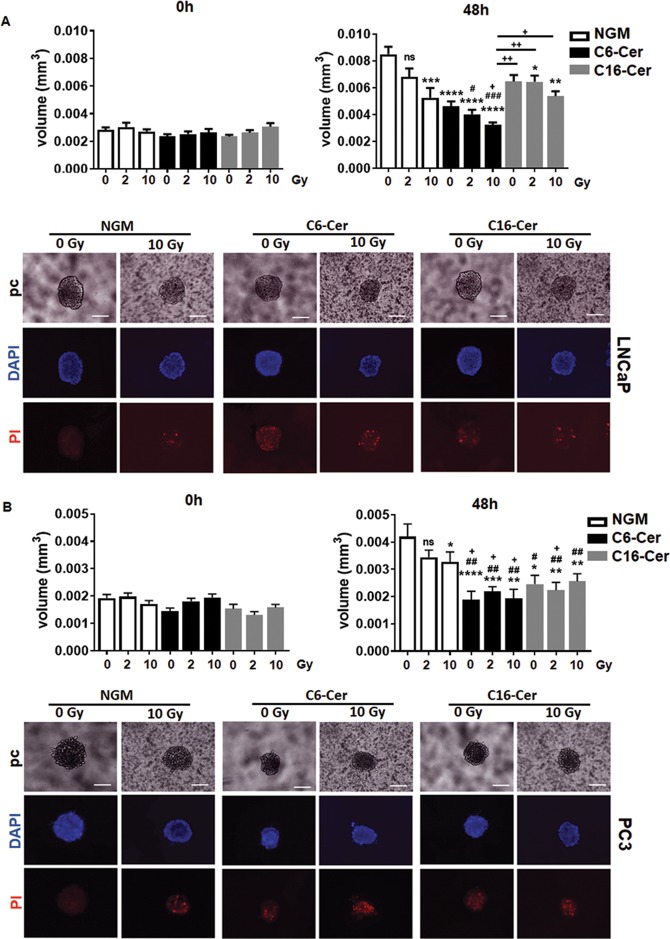
Exogenous ceramide treatment induced PCa cell growth retardation and cell death. **a** LNCaP and **b** PC3 cells were cultured as spheroids and irradiated with 0 (control), 2, or 10 Gy in the presence of C6- and C16 ceramide (Cer) or vehicle control (in normal growth media, NGM). Pictures were taken at the time of irradiation (0 h) and 48 h later. Scale bar represents 50 µm. Representative phase-contrast images from three individual experiments are shown. Graphs depict spheroid volumes as determined from three independent experiments (*n* = 3, SEM) where at least 10–15 spheroids per condition each were measured. *p < 0.05, **p < 0.01, ***p < 0.005, ****p < 0.001 (compared with 0-Gy NGM); ^#^*p* < 0.05, ^##^*p* < 0.01, ^###^*p* < 0.005 (compared with 2-Gy NGM); ^+^*p* < 0.05, ^++^*p* < 0.01 (compared with 10-Gy NGM) by two-way ANOVA followed by post hoc Tukey’s test. Cell death was analyzed afterward by fluorescence microscopy using propidium iodide. Representative phase-contrast (pc) images and simultaneously recorded fluorescent photographs (0 and 10 Gy; 48-h time point) from three individual experiments are shown. Scale bar represents 25 µm.

We then investigated, if and how radiation of EC with a differential CAV1 content would affect the radiation response of adjacent PCa cells. Therefore, ECs were directly co-cultured with LNCaP and PC3 cells using spheroids embedded in Matrigel and subjected to RT (Fig. 4). LNCaP cells (low endogenous CAV1) co-cultured with CAV1-deficient EC showed a tendency toward accelerated growth under control conditions (Fig. 4a). RT then induced a significant growth delay of the spheroids containing LNCaP cells and CAV1(−) EC. In contrast, IR treatment of cocultures of LNCaP with CAV1-proficient EC did not affect growth inhibition. A similar increased radiation response was observed for 3D spheroids containing CAV1-deficient EC and PC3 cells (high endogenous CAV1) (Fig. 4b). In both PCa cell types, the reduction of spheroid growth was accompanied by the presence of tumor cells that were permeable for propidium iodide, and thus clearly showed radiation-induced cell death (Fig. 4a, b). Moreover, the direct contact between EC and tumor cells seemed to be important, because cultivation of PCa cells with conditioned medium collected from CAV1-proficient or -deficient EC was not sufficient to induce cell death (Supplemental Fig. S4). Conclusively, we showed here that a reduction of endothelial CAV1 not only impacts on the EC radiation response itself resulting in EC radiosensitization^19^, but rather leads to an increased radiation response of adjacent PCa cells, presumably through EC-derived ASMase/ceramide-mediated cell death induction. To provide a proof of principle, PCa cells were subjected to exogenous ceramide treatment (Fig. 5). LNCaP (Fig. 5a) as well as PC3 (Fig. 5b) cells were cultured as spheroids and left non-irradiated or were irradiated with 2 Gy (to include a more clinically relevant fractionated low-dose setting) or 10 Gy in the presence of C6- and C16 ceramide. Both ceramide species significantly reduced the respective spheroid growth, and resulted in cell death induction even without IR (Fig. 5a, b). For LNCaP cells, additional growth retardation was detected in combination with IR (Fig. 5a, b). Together with the results of the EC co-culture experiments, these data strongly account for targeting the ASMase/ceramide-mediated cell death as potential anti-cancer strategy, and in particular for improving the radiation response of tumors.

### CAV1-proficient, radio-resistant PC3 cells were characterized by increased ceramide levels

**Fig. 6:**
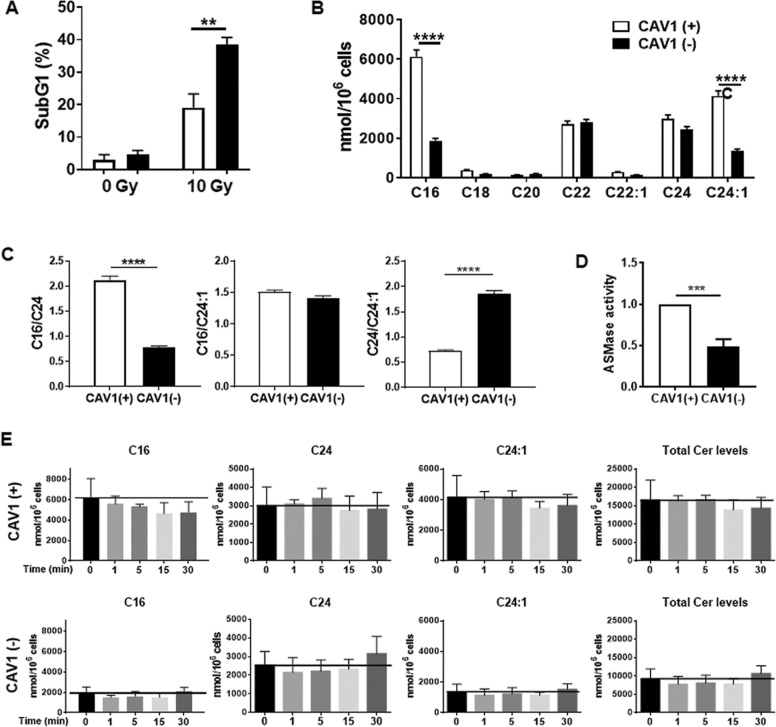
Radiation-resistant CAV1(+) PC3 cells are characterized by higher levels of total ceramide, which correlated with an increased ASMase activity. CAV1-proficient [CAV1(+)] and CAV1-deficient [CAV1(−)] PC3 PCa cells were subjected to radiation treatment (0-Gy control or 10 Gy). **a** Apoptotic cells (sub-G1) were analyzed by flow cytometry 48 h after irradiation. Graphs consist of data from 3 to 4 individual experiments (with SEM). **p < 0.01 by two-way ANOVA with post hoc Sidak’s multiple-comparison test. **b** Different expression of ceramide species was measured by LC–MS in CAV1(+) and CAV1(−) PC3 cells. Bars represent the mean of three individual experiments with SEM. Statistical analysis was performed using two-way ANOVA followed by post hoc Sidak’s test (****p < 0.001). **c** The ratios of saturated to unsaturated ceramide C16–C24:1 and C24–C24:1 were calculated in CAV1(+) and CAV1(−) PC3 cells. The results are shown of three individual experiments with SEM. Statistical analysis was performed using Student’s *t* test with Welch’s correction. *p* Value indicates ****p < 0.001. **d** ASMase activities of differential CAV1-expressing PC3 cells were measured at control conditions. Activities are shown in relation to CAV1(+) cells set as 1 (*n* = 4, SD). *p* Value indicates ***p < 0.001 and was analyzed by Student’s *t* test with Welch’s correction. **e** Timeline of indicated ceramide species as well as total ceramide levels generated by CAV1(+) and CAV(−) PC3 cells after IR treatment. Samples were taken 1, 5, 15, and 30 min after 10-Gy irradiation (*n* = 3, SD).

Similar to the reduction of CAV1 in EC, a reduction of CAV1 in PCa cells resulted in an increased radiosensitivity^37^ (Fig. 6a). We investigated the CAV1-dependent generation of ceramide species in either CAV1-proficient or CAV1-deficient PC3 cells analogously to the above analysis (Fig. 6). PC3 cells expressed C16, C22, C24, and C24:1. However, C16, C24, and C24:1 ceramide species were significantly increased in radio-resistant CAV1-expressing PC3 cells (Fig. 6b, c). As the chain length and saturation of biologically active ceramides serve as important regulatory factors for the regulation of cell death or survival, the increased levels of unsaturated C24:1 ceramide, and thus a respective decreased C24/C24:1 ratio in the radio-resistant CAV1(+) PC3 could already indicate the inhibition of apoptosis pathways (Fig. 6b, c). Accordingly, a significantly increased ASMase activity was observed in the CAV1-proficient PC3 cells (Fig. 6d). Of note, radiation did not affect the generation of ceramide (Fig. 6e). PC3 ceramide levels were generally higher than the CAV1-dependent ceramide levels observed in EC, and in particular levels of the C16 and C24:1 ceramide species (Supplemental Fig. S5). At the same time, the activity of ASMase is really low and not inducible upon IR (Supplemental Fig. S5 and not shown).

These findings suggest that the different levels of certain ceramide species induced in the respective cells with a differential CAV1 content, are decisive for its radiation sensitivity. Most importantly, the inducibility of the ASMase-dependent ceramide generation upon IR (as seen in EC) and the subsequent ceramide-mediated membrane remodeling seemed to be decisive for affecting the cell’s signaling and thus transmitting the radiation response. In contrast, the steady-state distributions of CAV1-affected ceramide levels in membranes seem rather to affect the ordering of the membrane and consequently membrane biophysics.

### Human advanced PCa specimen showed an increased ceramide immunoreactivity indicating radiation resistance

**Fig. 7:**
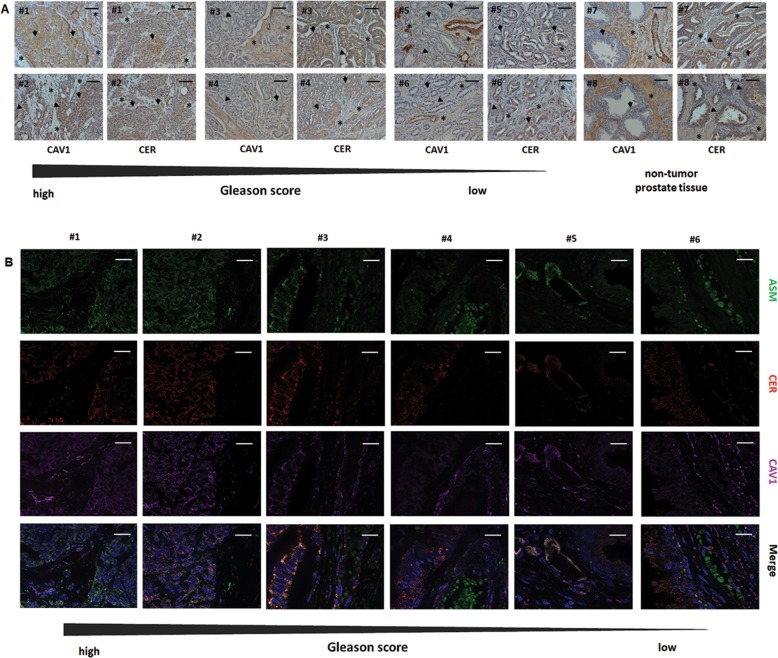
Immunohistological analysis of CAV1, ASMase, and ceramide expression levels in human PCa tissues. Paraffin sections of human PCas were stained for the indicated antibodies using either IHC (**a**) or immunofluorescence (**b**). Gleason grading scores were divided into low (Gleason Score ≥6, Grade group 1), intermediate (Gleason Score 7 (a/b), Grade groups 2 and 3), and high scores (Gleason Score ≥8, Grade groups 4 and 5). Asterisks mark stromal compartments and bold arrows point to epithelial structures. Sections were counterstained using hematoxylin (a, IHC) or DAPI (b, immunoflourescence). Representative images are shown. Magnification ×40 (phase contrast), scale bar: 100 µm; ×63 (immunofluorescence), scale bar: 20 µm.

As an increase in epithelial CAV1 (together with a loss of stromal CAV1) has been linked to PCa RT resistance^30,37^, we decided to explore a potential link between the levels of ceramide, ASMase, and CAV1 as well as their respective stromal–epithelial distribution, in tissue specimen of human PCa (Fig. 7). Ceramide and ASMase immunoreactivity seem to be increased in the CAV1-positive malignant epithelial cells of advanced PCa specimen. Furthermore, there was a trend toward a less intense staining for ceramide and ASMase in CAV1-deficient stromal compartments of tumor samples with higher Gleason grade. Of note, CAV1-expressing EC seemed to remain ceramide- and ASMase-positive upon tumor progression (Fig. 7). Though we were not able to distinguish the different ceramide species in tumor specimen, we used the MS analyses of the respective tumor cells and EC, as well as fibroblasts being either CAV1-proficient or -deficient to mimic the human situation with respect to the differential CAV1 levels being characteristic for low-grade tumors and advanced tumor stages (Supplemental Fig. 6). In addition to the increased unsaturated C24:1 ceramide species detected in the more radio-resistant CAV1(+) PC3 cells, CAV1-deficient fibroblasts, as found in advanced, more radio-resistant tumor stages, showed significantly upregulated C24:1 ceramide levels. These results suggest that the local concentrations of certain ceramide species as found in a complex mixture of cells like in a tumor were decisive for the regulation of cell death or survival. In particular, increased levels of very-long-chain and concomitantly unsaturated ceramides might scavenge the effects of apoptosis-inducing long-chain ceramides.

## Discussion

Ceramide-induced membrane remodeling following IR leads to the formation of LLP as effective signalosomes^38^. Here, we showed that endothelial CAV1 critically regulates this ASMase/ceramide-mediated response to IR. CAV1-enriched caveolae usually function as signaling organizing centers and platforms by modulating specific signals in a temporally and spatially controlled manner^39–41^. For example, the major angiogenic growth factor VEGFR2/KDR is partially localized in caveolae, and VEGFR2 binding to CAV1 significantly decreased its ability to become activated^41^. After stimulation, VEGFR2 shifts to non-raft membrane portions resulting in the activation of its tyrosine kinase^41^. These observations match with our previous findings about an increased angiogenic phenotype of CAV1-deficient EC^19^. In mature functional blood vessels, p38 activation and HSP27 phosphorylation are normally suppressed^42^. VEGFR2 was even shown to act upstream of p38-mediated HSP27 phosphorylation^42^. We further show that these apoptosis-prone ECs were characterized by increased levels of phosphorylated HSP27 as well as decreased AKT levels. The ceramide-mediated inhibition of AKT activation was already reported^36,43^. Pro-apoptotic stress-induced ceramide fostered apoptosis via the downregulation of the PI3K/AKT survival pathway^36^. A downregulation of AKT was further reported to increase cellular stress in EC via activation of p38^44^.

**Fig. 8:**
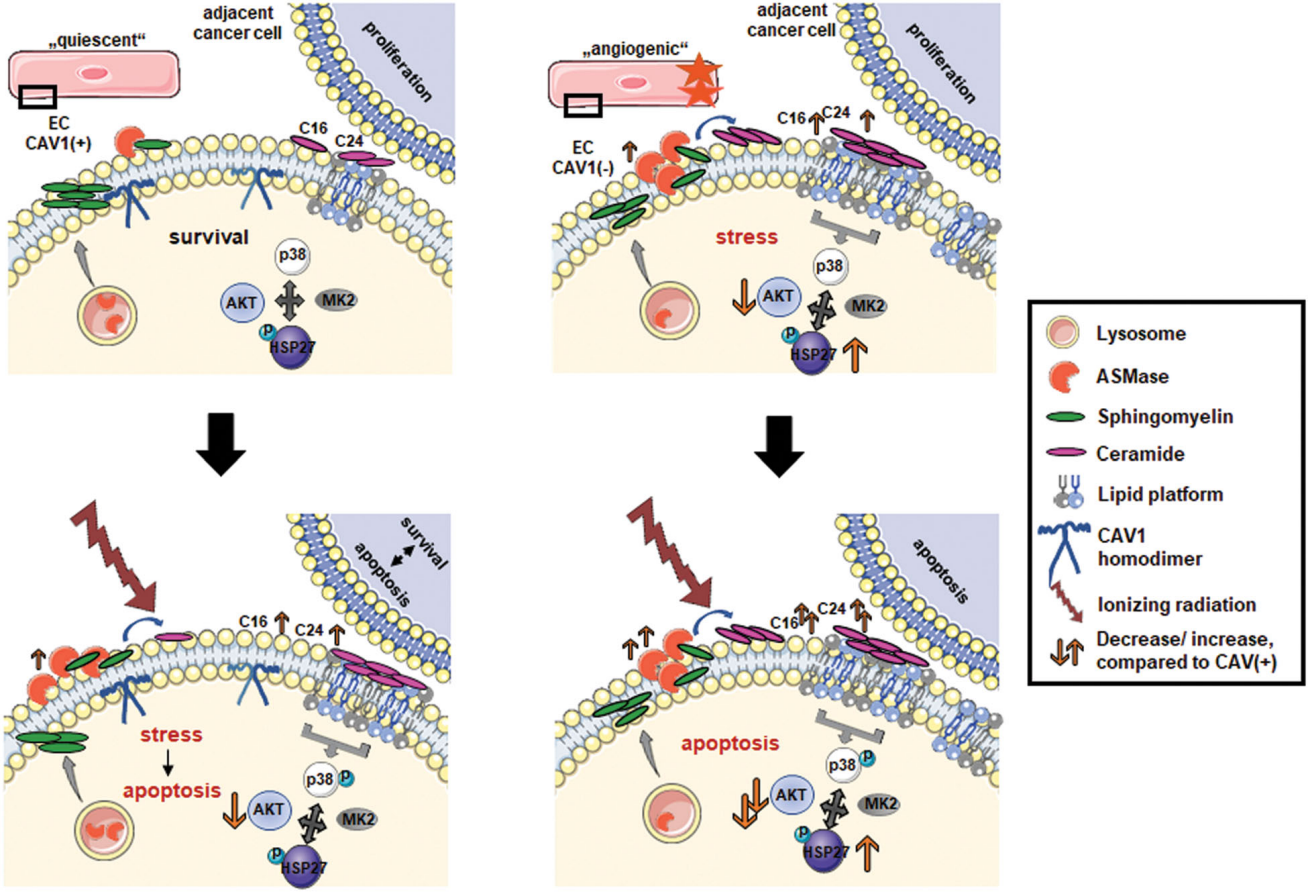
Schematic overview of CAV1-dependent ASMase/ceramide signaling in EC. Under normal (steady-state) conditions, ASMase is located in lysosomes (CAV1-proficient EC, upper panel, left). Upon stress induction by ionizing radiation (IR), ASMase is translocated to the plasma membrane, presumably facilitated by CAV1-abundant regions (lower panel, left). Thereby, IR leads to a rapidly increased ASMase activity, followed by ceramide generation (specifically of C16 and C24), which goes along with formation of large lipid platforms, and altered signal transduction directly affecting the p38/HSP27/MK2/AKT axis. The IR-induced remodeling of plasma membrane prior signaling implicates that CAV1 and ASMase-dependent ceramide generation are essential for stress-responsive cell-fate decisions like survival and/or cell death. CAV1 downregulation or absence in EC caused a constitutively upregulated ASMase activity that facilitates the increased generation of ceramide and specifically of the ceramide species C16 and C24 (upper panel, right). The resulting increase in (stable) large lipid platform formation permanently alters then the ceramide-dependent signaling of the p38/HSP27/MK2/AKT axis pathway finally priming EC apoptosis. Upon IR-induced stress, increased ceramide levels further increased membrane remodeling resulting in an amplified LLP/signalosome signaling finally leading to increased HSP27 activation, AKT deactivation, and then EC apoptosis (lower panel, right). Elevated ASMase/ceramide signaling in CAV1(−) EC might impact on adjacent tumor cell’s survival and growth in a negative manner.

However, how a loss of CAV1 in EC affects downstream signaling and/or exerts paracrine effects on adjacent parenchyma remained elusive^45^. We showed now that CAV1 deficiency in EC increased the formation of membrane signalosomes and thus affected p38 and AKT downstream signaling ultimately leading to EC apoptosis (Fig. 8). High doses of IR induced ceramide generation from sphingomyelin hydrolysis by the translocation and subsequent activation of the lysosomal ASMase to the plasma membrane^25,27^. Due to their specific biophysical properties, ceramides fostered then the formation of ceramide-enriched LLP, which acts as signaling platforms for an altered signaling and apoptosis initiation. Ceramide is known to displace cholesterol from lipid rafts and to decrease the association of cholesterol to CAV1^40^. Mechanistically, a signaling complex consisting of AKT, p38, MK2 (MAPKAPK-2), and HSP27 was concluded^46,47^. In unstressed situations, p38 constitutively interacts with HSP27. MK2 is needed for the formation of the module together with AKT^46^. Upon cellular stress, AKT and p38 were activated and disrupted the interaction between p38 and HSP27 by phosphorylation of HSP27^46^. Upon activation, HSP27 was shown to dissociate from that complex and in particular from AKT^47^. The disruption of the HSP27–AKT interactions on the other hand impaired AKT activation. Sequestration of HSP27 from AKT increased apoptosis rates^47^. Thus, through control of AKT activity, HSP27 regulates apoptosis. In addition, MK2 (activated by p38) was shown to phosphorylate AKT. Conclusively, plasma membrane remodeling prior signaling implicates that CAV1 and ASMase-dependent generation of ceramide were essential for stress-responsive cell-fate decisions like survival and/or cell death.

One of our initial hypotheses was that lowering the CAV1 content, particularly in EC, might increase the radiation response of tumors with increased CAV1 expression levels^19^. Silencing of CAV1 in cancer cells themselves was already shown to re-sensitize tumor cells to RT-induced apoptosis^48–51^. Even in PCa, which is characterized by increased CAV1 expression levels in malignant epithelial cells at advanced, more radio-resistant disease stages, a reduction of CAV1 was suited to achieve tumor cell radiosensitization^30,37^. We demonstrated here that RT induced a significant growth delay and cell death in PCa spheroids when co-cultured with CAV1-deficient ECs that were at least partially mediated by RT-induced ASMase/ceramide signaling in EC. Quantification of the different ceramide species using LC–MS/MS revealed that EC expressed C16, C24, and C24:1 ceramide differentially, particularly in response to IR. CAV1(−) EC showed a decreased C24/C24:1 ceramide ratio, which, together with elevated C16 ceramide levels, might (i) account for the increased sensitivity of those cells to radiation-induced apoptosis, and (ii) might alter the behavior, including the radiation response of adjacent PCa cells. Of note, EC apoptosis by the production of ceramide was shown to depend on doses higher than 5 Gy^52–54^. Although we did not investigate here the potential effects of EC on adjacent PCa cells at doses lower than 10 Gy, the treatment of PCa spheroids with different exogenous ceramide species revealed a significant growth retardation and cell death induction.

In the complex in vivo situation of a tumor, the RT-induced potential of EC to induce cancer cell apoptosis may not be sufficient for the overall therapy response because of the tumor’s complex microenvironment. We additionally investigated the CAV1-dependent expression of ceramide species in PCa cells and fibroblasts with differential CAV1 levels. Although C16, C22, C24, and C24:1 were shown to be expressed in both PC3 variants, the C16 and C24:1 ceramide species were significantly increased in the more radio-resistant CAV1-expressing PC3 PCa cells. C24:1 ceramide was even increased in radio-resistant CAV1-deficient fibroblasts. IR-induced activation of apoptosis preferentially facilitated ceramide-enriched membrane domains that were formed by saturated ceramides^55,56^. The increase in unsaturated very-long-chain C24:1 ceramide in both cell types here accounted for an increased C24/C24:1 ratio, which might counteract the assumed apoptosis-inducing effects of long-chain ceramides.

Thus, the overall concentration and distribution of certain ceramide species might be crucial for apoptosis induction^57^. We showed here that advanced PCa specimens were characterized by increased ceramide as well as ASMase amounts in the CAV1-positive malignant epithelial cells. Using CAV1(+) EC, CAV1(−) PC3 together with CAV1(+) HS5 fibroblasts as a model combination for the CAV1 status of the respective cells found in healthy or low-grade prostate carcinoma, and CAV1(+) EC, CAV1(+) PC3 together with CAV1(−) fibroblasts as a model combination for advanced tumor stages, it becomes clear that the very-long-chain ceramides are expressed in higher amounts in each respective more radio-resistant CAV1 variant. The increased levels of unsaturated C24:1 in CAV1(+) PC3 and CAV1(−) fibroblasts in turn may account then for the absent apoptosis usually mediated by long-chain ceramides, in particular by C16 ceramide derived from irradiated CAV1(+) EC. It is important to note that, in contrast to the CAV1-dependent ceramide levels in EC, the differential ceramide levels and the respective ceramide species detected at steady state in CAV1 pro- and deficient PCa cells and stromal fibroblasts were not further affected by IR treatment. Accordingly, modifying the equilibrium between the chain lengths of ceramides, e.g., a shift from C24 to C16 was shown to increase the susceptibility of cancer cells to apoptosis^58^. In PCa, increases in C16 ceramide levels fostered apoptosis of LNCaP cells^59^.

Conclusively, targeting the membrane’s lipid organization and/or composition here bears the potential for a therapeutical approach. Ceramides or synthetic, metabolically stabilized analogs with pro-apoptotic properties (e.g., C16) may be useful as anticancer agents^57^. In PCa, the use of agents that elevate ceramide levels as novel chemotherapeutic agents was already suggested^60^. Increases in cellular ceramide here resulted in cell death induction, and in particular radiosensitization of the respective PCa tumors^60^. These studies, together with the results we presented here, suggest that targeting the ceramide pathways may be a novel treatment strategy for radiation-resistant PCa.

## Materials and methods

### Materials

The CAV1 antibody was from Santa Cruz Biotechnology (Santa Cruz, CA, USA); antibodies against phospho-p38 MAPK (Thr180/Tyr182), p38, phospho-HSP27 (Ser82), HSP27, HSP90, phospho-AKT (Ser473), and AKT were purchased from Cell Signaling Technology (Danvers, MA; all 1:1000 WB). Antibody for beta-actin (clone AC-74, A2228) was from Sigma-Aldrich (St. Louis, MO, USA). The goat-anti-ASMase antibody was kindly provided by Prof. K. Sandhoff (Bonn, Germany)^61^.

### Cell cultures

The human PCa cell lines LNCaP and PC3 as well as the fibroblast cell line HS5 were cultured in RPMI 1640 medium (Gibco, ThermoFisher, Waltham, MA, USA) supplemented with 10% fetal calf serum (FCS) and 100 U penicillin/streptomycin (Sigma-Aldrich, St. Louis, MO, USA) at 37 °C, 5% CO_2_, and 95% humidity. All cells were routinely tested for mycoplasma contamination (every two weeks) and periodic authenticated by STR profiling (if necessary, no later than yearly). ECs AS-M5 were cultured in Medium 199 (Gibco, ThermoFisher, Waltham, MA, USA) supplemented with 20% FCS, 100 U penicillin/streptomycin, and 15 mg of EC Growth Supplement (Sigma) at similar conditions. Cells were passaged 2–3 times per week. CAV1 levels in AS-M5, HS5, and PC3 cells were downregulated by lentiviral-transduced shRNA as previously described and labeled with green-fluorescent protein^19,37^. Exogenous C6- and C16 ceramide (Cayman Chemical, Ann Arbor, MI, USA) was solved in 100% EtOH and added after radiation treatment (both 10 µM). Prior to treatment, cells were starved in medium containing 0.5% FCS overnight. The selective inhibitor of p38 MAPK SB203580 (4-(4-fluorophenyl)-2-(4-methylsulfinylphenyl)-5-(4-pyridyl)-imidazole, Cell Signaling Technology) that inhibits p38 MAPK catalytic activity by binding to the ATP-binding pocket, and thus the activation of MAPKAPK-2 and subsequent phosphorylation of HSP27, was dissolved in DMSO and used at 10 µM 2 h prior irradiation (end concentration).

### Spheroid culture

PC3 or LNCaP cells were cultured alone or in combination with AS-M5 CAV1(+) or CAV1(−) cells in hanging drops for 24 h (ratio 1/1). Afterward, spheroids were plated in normal growth medium (NGM) with growth factor-reduced Matrigel (Corning, NY, USA) (dilution: 1/2). Pictures were taken directly and 48 h after treatment at ×10 magnification. Size was measured and calculated using ImageJ software. For the detection of cell death, spheroids were incubated thereafter for additional 15 min with propidium iodide (50 µg/mL) and DAPI (4′,6-diamidino-2-phenylindole) for nuclei staining and analyzed by fluorescent and phase-contrast microscopy.

### Irradiation

Irradiation of cultured cells was performed using an Isovolt-320-X-ray machine (Seifert-Pantak) at 320 kV, 10 mA, and a 1.65-mm aluminum filter at a distance of 50 cm. The dose rate was approximately 3 Gy/min with an energy of the tube of 90 kV (~45-keV X-rays). Irradiation for LC–MS/MS, ASMase activity, and LLP staining was performed with a 160-kV irradiator (CP160 Faxitron X-ray, USA) at a dose rate of 1.48 Gy/min.

### siRNA transfection

Control A and B siRNA, and HSP27 siRNA (h; sc-29350) were from Santa Cruz Biotechnology. For siRNA treatment, CAV1(+) and CAV1(−) EC were transfected with non-silencing (control) and HSP27 siRNA using Santa Cruz Biotechnology’s siRNA Transfection Reagent according to the manufacturer’s instructions. Cells were irradiated the next day with 0 (−) or 10 (+) Gy and analyzed for downstream effects after additional 48 h (72 h after transfection).

### Conditioned medium

EC cells were grown until 80% confluency in NGM, irradiated with 0 Gy (control) or 10 Gy, and cultured for an additional 48 h in low FCS medium (2%). The supernatant was harvested and centrifuged to separate dead cells. Conditioned medium was used with NGM (ratio 1:2).

### Flow cytometry

Apoptotic cells (sub-G1 fraction) and cell cycle phases were analyzed by using Nicoletti/propidium iodide staining [PI, 0.1% sodium citrate (w/v), 50 µg/mL PI (v/v), and 0.05% Triton X-100 (v/v)] as previously described^62^. Cells were stained for 30 min in the dark and subsequently measured with flow cytometer FACS Calibur (BD, Heidelberg, Germany, FL2). Analysis was done using FlowJo software.

### LC–MS for ceramide quantification

Extraction of lipids was performed by using chloroform/methanol (1/2, v/v) and LC–MS/MS was done as previously described^63^. The assay was performed on a HP 1100 series liquid chromatography (Agilent) in line with an electrospray ion mass spectrometer Esquire 4.5 series (BD). Different ceramide species were analyzed with an UptiSphere 5ODB column and the integration of the respective species was done using QuantAnalysis software (BD). Samples contained an internal standard (C17:1) that was used as a loading control.

### ASMase activity assay

ASMase activity was quantified with BODIPY Sphingomyelin (Invitrogen). One microgram of lysed protein sample was incubated with fluorescent sphingomyelin overnight at 37 °C allowing ASMase cleavage to BODIPY ceramide. The reaction was stopped using a mixture of chloroform/methanol (1/1 v/v), and after centrifugation, the organic phase containing the lipids was evaporated with nitrogen. Separation of the lipids was performed using a thin-layer chromatography silica membrane in chloroform/methanol (95/5, v/v) (Whatman, UK). After drying, the plate was analyzed with chemoluminescence, and ASMase activity was determined from the relation of BODIPY ceramide to sphingomyelin.

### Western blotting

After treatment, cells were scraped in ice-cold RIPA buffer (150 mmol/L NaCl, 1% NP40, 0.5% sodium deoxycholate, 0.1% sodium dodecylsulfate, 50 mmol/L Tris/HCL, pH 8, 10 mmol/L NaF, and 1 mmol/L Na_3_VO_4_) containing complete Protease inhibitor cocktail (Roche). Two to three thaw/freeze cycles were performed before subjecting whole-cell lysates to SDS-gel electrophoresis as previously described^19,37,64^.

### Large lipid platform (GM1) staining

Cells were seeded on coverslips, irradiated, and placed directly on ice at the indicated time points after IR. Lipid platforms were stained by using 0.1 mM AlexaFluor647-conjugated cholera-toxin B-subunit for 45 min as described before^25^. Cells were fixed with 4% PFA for 20 min. After blocking with 10% bovine serum albumin for 20 min, co-staining with CAV1 was performed in a dilution of 1:100 in blocking buffer for 1 h. Samples were mounted with GoldProlong containing DAPI (ThermoFisher) to visualize nuclei. Pictures were taken with a Leica confocal microscope.

### Immunofluorescent detection of double-strand breaks

Cells were seeded on coverslips and treated with 3-Gy irradiation. Fixation was performed at the indicated time points (0.5–24 h) with 4% PFA for 15 min following permeabilization with 0.1% Triton for 5 min. Staining of ɣH2.AX foci was done using an AlexaFluor647-labeled anti-H2AX antibody conjugated with (BD) for 1 h. Samples were incubated with Hoechst 33342 to visualize nuclei. At least 50 foci per condition were counted using the focinator software^65^.

### Real-time (RT) qPCR

RNA was isolated by using RNeasy Mini Kit (Qiagen, Hilden, Germany) after the manufacturer’s instructions. One microgram of cDNA was used, and expression levels of the indicated genes were compared with housekeeping gene beta-actin (set as 1). The following primer sequences were used: CerS1 for CCTCCAGCCCAGAGAT, rev: AGAAGGGGTAGTCGGTG; CerS2 for CCAGGTAGAGCGTTGGTT, rev: CCAGGGTTTATCCACAATGAC; CerS3 for CCTGGCTGCTATTAGTCTGAT, rev: TCACGAGGGTCCCACT; CerS4 for GCAAGGATTTCAAGGAGCAG, rev: AACAGCAGCACCAGAGAG; CerS5 for CAAGTATCAGCGGCTCTGT, rev: ATTATCTCCCAACTCTCAAAGA; CerS6 for AAGCAACTGGACTGGGATGTT, rev: AATCTGACTCCGTAGGTAAATACA; β-ACTIN for TCCATCATGAAGTGTGACGT, rev: GAGCAATGATCTTGATCTTCAT. RT-qPCR was performed in an Agilent Aria cycler.

### Human tumor tissue

Tissues from human prostate carcinomas were obtained during surgery according to local ethical and biohazard regulations. All experiments were performed in strict accordance with local guidelines and regulations. Resected tissue specimens were processed for pathological diagnostic routine in agreement with institutional standards, and diagnoses were made based on current WHO and updated ISUP criteria^19,37^. All studies including human tissue samples were approved by the local ethics committee (Ethik-Kommission) of the University Hospital Essen (Nr. 10-4363 and 10-4051). Human tissue samples were analyzed anonymously. Immunohistochemistry (IHC) and immunofluorescence staining was performed on formalin-fixed and paraffin-embedded slides of human PCa samples as previously described^30,37^. Samples were prepared by using a descending alcohol series and incubation with target retrieval solution (DAKO). Afterward, slides were blocked with a 2% FCS/PBS blocking solution to reduce unspecific interactions, and primary antibodies were incubated overnight at 4 °C. Antigens were detected either by horseradish-peroxidase conjugated secondary antibodies and DAB staining (IHC) or fluorescently labeled secondary antibodies. Nuclei were counterstained with hematoxylin or DAPI.

### Statistical analysis

If not otherwise indicated, data were obtained from at least three independent experiments (*n* = 3). Mean values were calculated and used for analysis of standard deviation or standard error. Statistical significance was evaluated by one- or two-way ANOVA followed by Tukey’s, Sidak’s, or Bonferroni multiple comparisons post test as indicated. Statistical significance was set at the level of *p* ≤ 0.05 (*p ≤ 0.05, **p ≤ 0.01, ***p ≤ 0.005, ****p ≤ 0.001). Data analysis were performed with Prism 5.0 software (GraphPad, La Jolla, CA, USA).

## Supplementary information

Supplemental Figure Legends

Supplemental Figure S1

Supplemental Figure S2

Supplemental Figure S3

Supplemental Figure S4

Supplemental Figure S5

Supplemental Figure S6
