# Supplementary material for: Caveolin-1 regulates the ASMase/ceramide-mediated radiation response of endothelial cells in the context of tumor–stroma interactions
Source: Cell Death Dis. 2020 Apr 9;11(4):228. doi: 10.1038/s41419-020-2418-z (PMC7145831; doi:10.1038/s41419-020-2418-z)
Supplement: Supplementary file 1 — Supplemental Figure Legends [file 41419_2020_2418_MOESM1_ESM.docx]

**Supplementary Figure Legends**

**Supplementary Figure S1**

**Silencing of HSP27 in cultured endothelial cells did not affect the cell cycle and p38/MAPK or AKT/PKB signals.**

CAV1-proficient [CAV1(+)] and CAV1-deficient [CAV1(-)] endothelial cells were transfected with control (non-silencing) or HSP27 siRNA and 24 hours later subjected to radiation treatment (0 Gy control or 10 Gy). (**A**). Whole cell lysates were used for Western blot analysis of the p38/MAPK and AKT/PKB pathways. Beta actin was used as a loading control. Representative blots of 4 individual experiments are shown. (**B**) Cell cycle phases and apoptotic cells (subG1) were analyzed by flow cytometry 48 hours after irradiation. Graphs consist of data from 4 individual experiments (with SEM). CAV1-proficent EC showed increased apoptosis levels by tendency upon HSP27 knockdown and radiation treatment. P-value as indicated. Radiation significantly reduced the CAV1(-) cell numbers in G1/G0 and significantly increased CAV1(-) EC in G2/M (**** p<0.001 for each phase by two-way ANOVA with post-hoc Tukey multiple comparison test; 0 Gy versus 10 Gy; not depicted), which was not further affected upon HSP27 silencing.

**Supplementary Figure S2**

**Differential ceramide levels of CAV1-proficient and -deficient EC.**

(**A**) Overview of all detected ceramide species by LC-MS in CAV1(+) and CAV1(-) EC (n=3, SEM). Statistical analysis was done by using two-way ANOVA followed by post-hoc Tukey’s multiple comparison. (**B**) The ratio of ceramide species C16 to C24, C16 to C24:1 and C24 to C24:1 was calculated in AS-M5 CAV1(+) and CAV1(-) cells. Control levels were pooled out of three individual experiments (n=3, SEM). Statistical significance was calculated with Tukey’s student t-test with Welch’s correction. P-value indicates *** p<0.001, **** p<0.0001. (**C**) RT-qPCR was performed using primers of the six known ceramide synthases (CerS1 – CerS6) in control (0 Gy) and 10 Gy irradiated AS-M5 CAV1(+) and CAV1(-) cells 48 hours after irradiation (n=4, SEM). Statistical analysis was done by using two-way ANOVA. P-value indicates * p<0.05.

**Supplementary Figure S3**

**The DNA damage response was not affected in** **CAV1(+) and CAV1(-) EC upon radiation treatment.** (**A**) Whole cell lysates were used for Western blot analysis of the p38/MAPK and AKT/PKB pathways by detecting the indicated involved proteins in control (0 Gy) and 10 Gy irradiated EC 5 min and 30 min after treatment. CAV1 expression levels were measured at the same time points. β-ACTIN was used as a loading control. Representative blots of 3-4 individual experiments are shown. (**B**) Representative images of H2A.X phosphorylation in CAV1(+) and CAV1(-) EC are shown at 0.5 h and 24 h timepoints after 3 Gy irradiation (upper panel). Scale bar: 5 µm. ɣH2A.X foci in AS-M5 CAV1(+) and CAV1(-) cells were counted after 3 Gy irradiation at the indicated timepoints (lower panel). Graph shows foci mean of three individual experiments with SEM.

**Supplementary Figure S4**

**Paracrine signaling of EC with a differential CAV1 expression is not sufficient to induce prostate cancer cell apoptosis.** Prostate cancer cells were subjected to radiation treatment (0 Gy control or 10 Gy) and subsequently cultivated with supernatant derived from CAV1(+) or CAV1(-) endothelial cells for additional 48 h. Cell cycle phases and apoptotic cells (subG1) were analyzed by flow cytometry. Graphs consist of data from three individual experiments with SD shown.

**Supplementary Figure S5**

**Most prominent ceramide species were significantly higher expressed in radio-resistant CAV1(+) PC3 cells compared to EC.** (**A**) All detected ceramide species as well as total ceramide levels (**B**) in CAV1-proficient and -deficient EC and PC3 cells, as analyzed by LC-MS (as depicted in recuded species in Figure 1 and 6), were compared next to each other. *<p 0.05 ****<p 0.001 by two-way ANOVA followed by Tukey’s test. (**C**) ASMase enzymatic activities were also compared. Activities are shown in relation to CAV1(+) EC, that was set at 1. **< p 0.01 ****<p 0.001 by one-way ANOVA followed by Tukey’s test. (**D**) Comparison of ceramide synthases (CerS1 – CerS6) expression levels (n=4, SEM). * p<0.05 by one-way ANOVA followed by Tukey’s test.

**Supplemental Figure S6**

**Overall ceramide levels of differential CAV1-expressing tumor and stroma cells in advanced prostate cancer were elevated.** (**A**) The schematic overview emphasizes CAV1 levels in stroma and epithelial/tumor cells in each stage. (**B**) Ceramide species of the healthy situation or low graded tumor using CAV1(+) EC, CAV1(-) PC3 and CAV1(+) HS5 cells as model with respect to the tumor’s CAV1 levels and distribution, as well as the CAV1 levels present in advanced prostate cancer [CAV1(+) EC, CAV1(+) PC3 and CAV1(-) HS5] were depicted as analyzed by LC-MS. As ceramides or combinations of the different ceramide species have even been proposed as biomarkers, LC-MS analysis indirectly revealed that C16:0, C24:0, and C24:1 levels were elevated in advanced PCa. Mean of three individual experiments are shown with SEM. Ratios of C16 to C24:1 and C24 to C24:1 in the healthy and advanced situation of prostate cancer are described. Ratios were taken out of three individual experiments and are shown with SEM.
