# Supplementary figures and images for: Caveolin-1 regulates the ASMase/ceramide-mediated radiation response of endothelial cells in the context of tumor–stroma interactions
Source: Cell Death Dis. 2020 Apr 9;11(4):228. doi: 10.1038/s41419-020-2418-z (PMC7145831; doi:10.1038/s41419-020-2418-z)

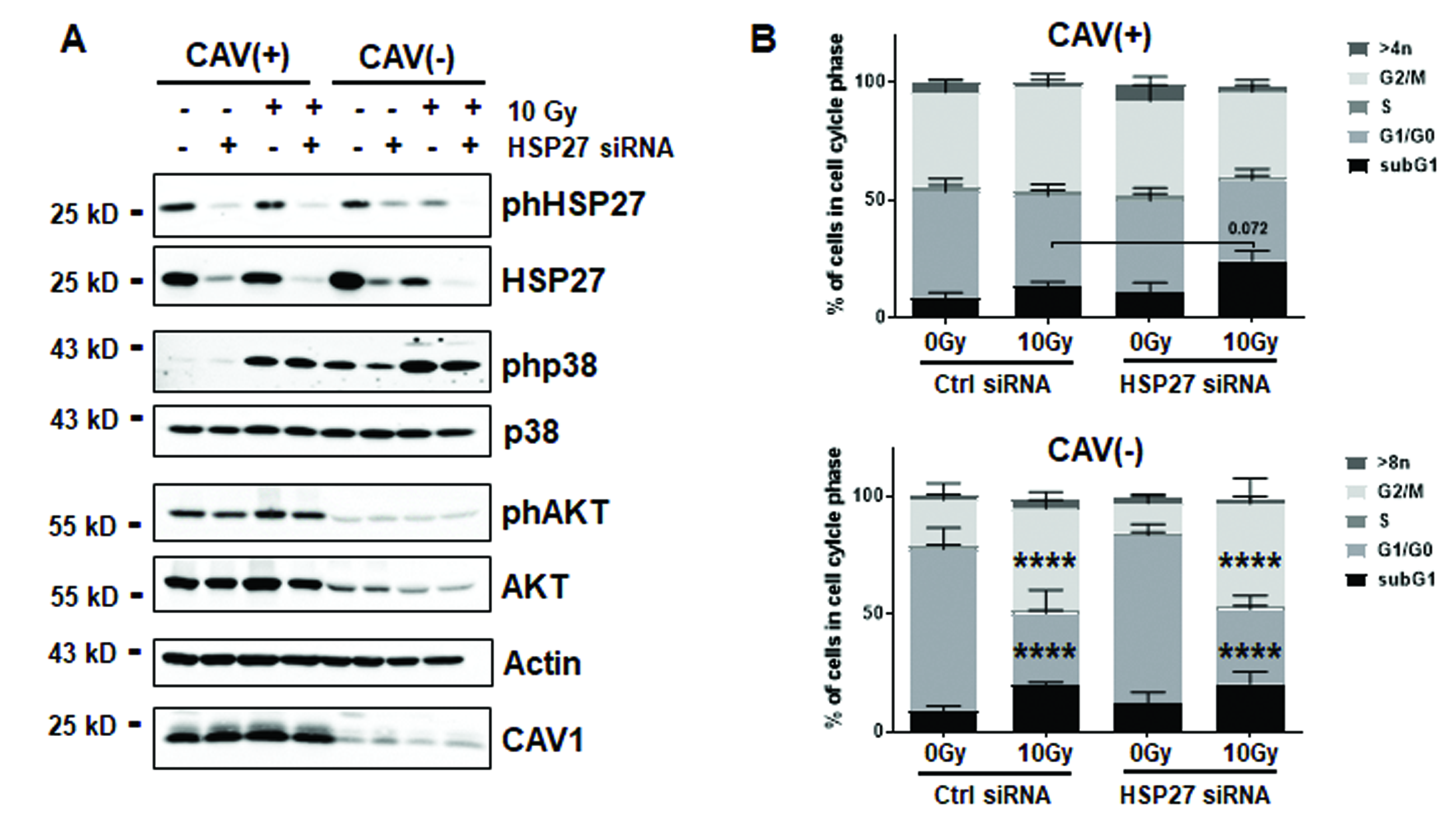

Supplement: Supplementary file 2 — Supplemental Figure S1 [file 41419_2020_2418_MOESM2_ESM.tif]

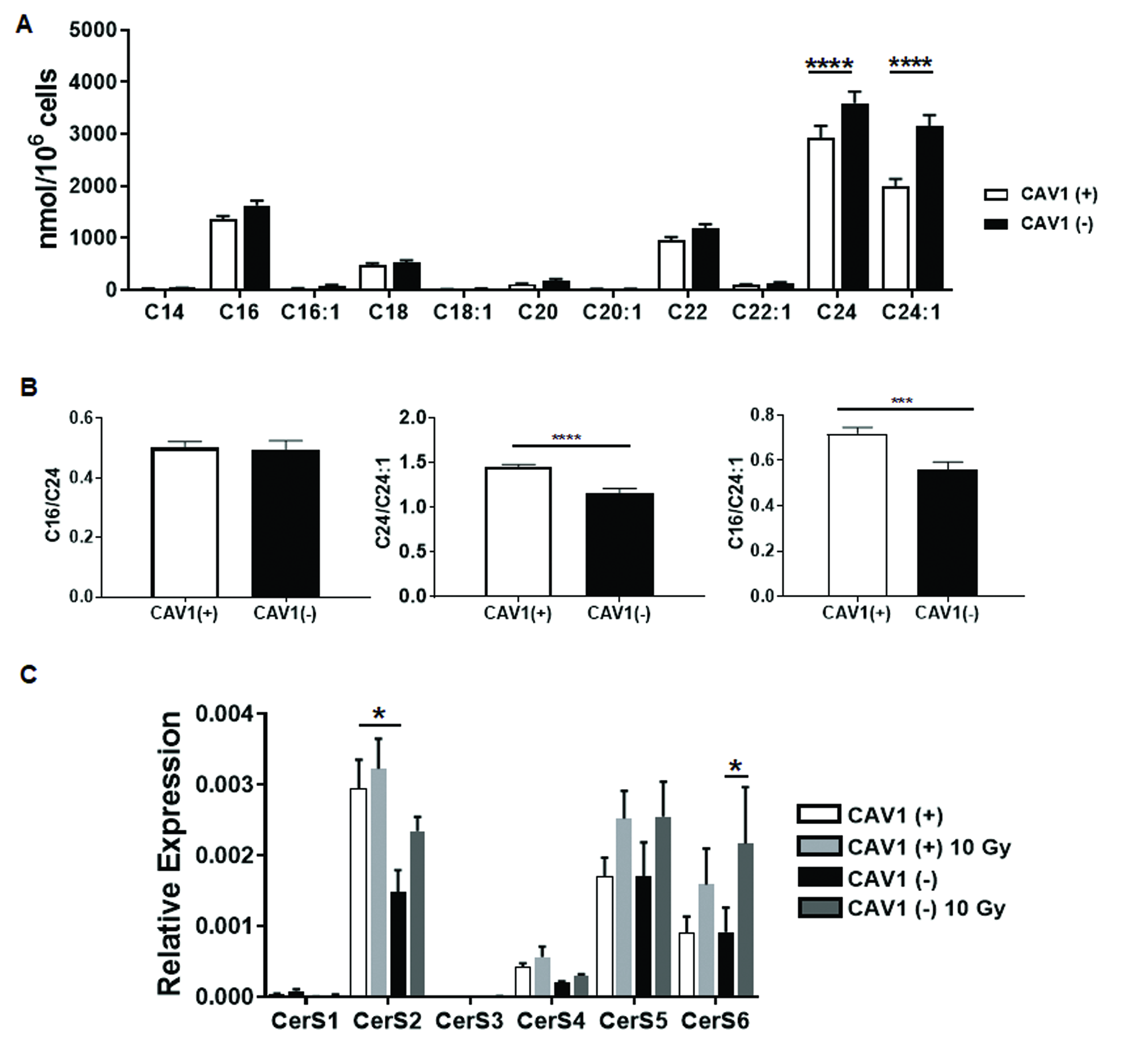

Supplement: Supplementary file 3 — Supplemental Figure S2 [file 41419_2020_2418_MOESM3_ESM.tif]

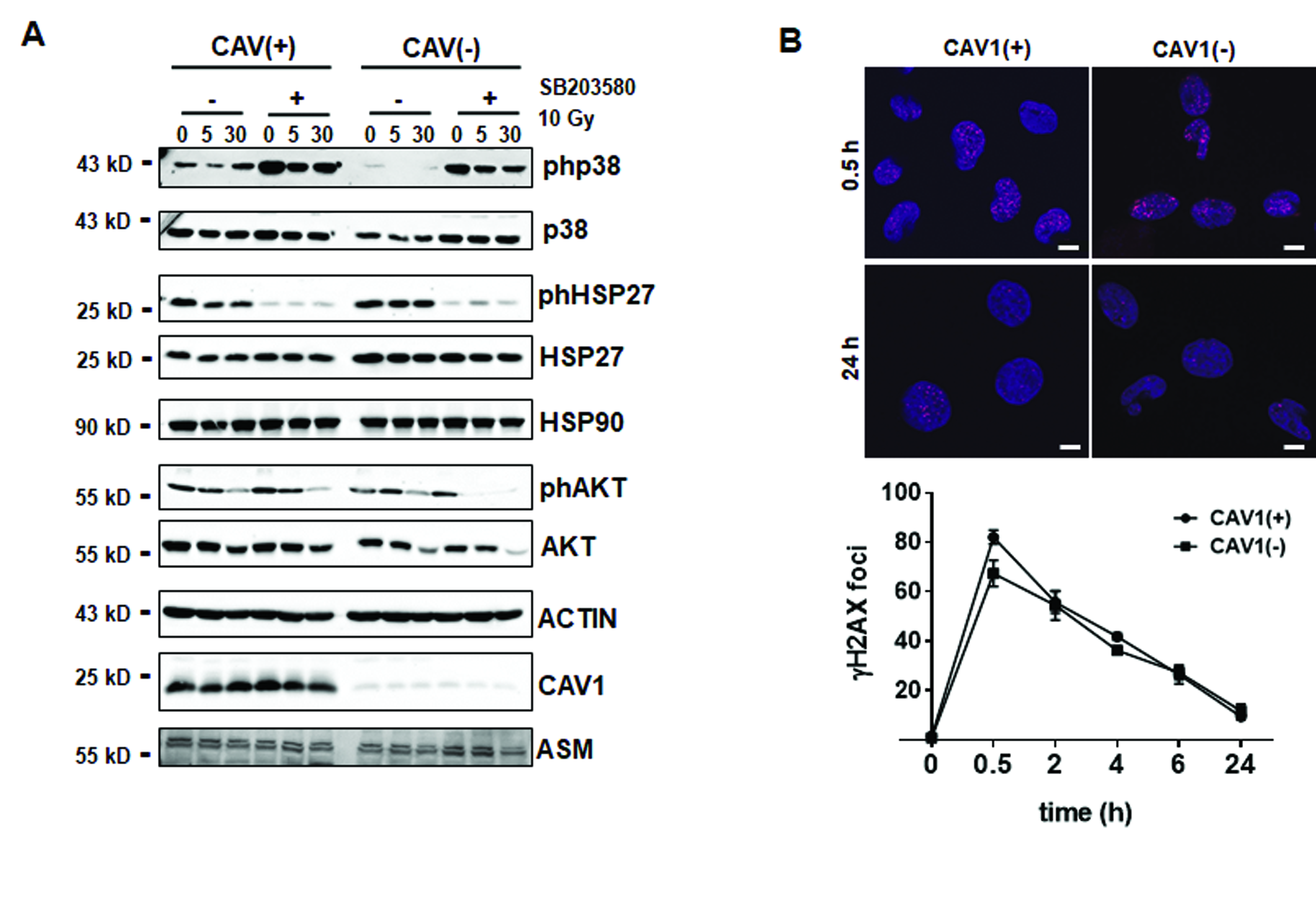

Supplement: Supplementary file 4 — Supplemental Figure S3 [file 41419_2020_2418_MOESM4_ESM.tif]

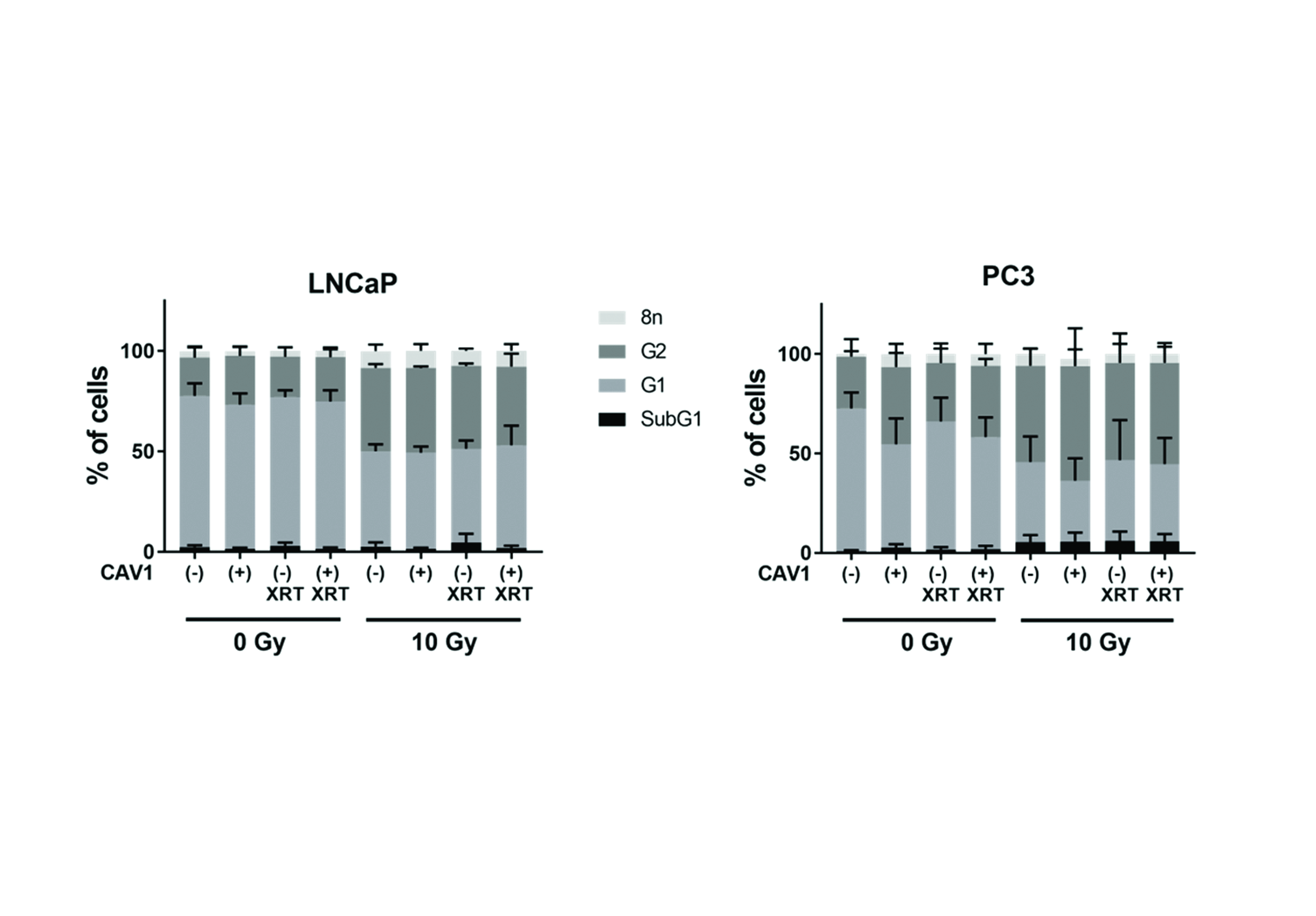

Supplement: Supplementary file 5 — Supplemental Figure S4 [file 41419_2020_2418_MOESM5_ESM.tif]

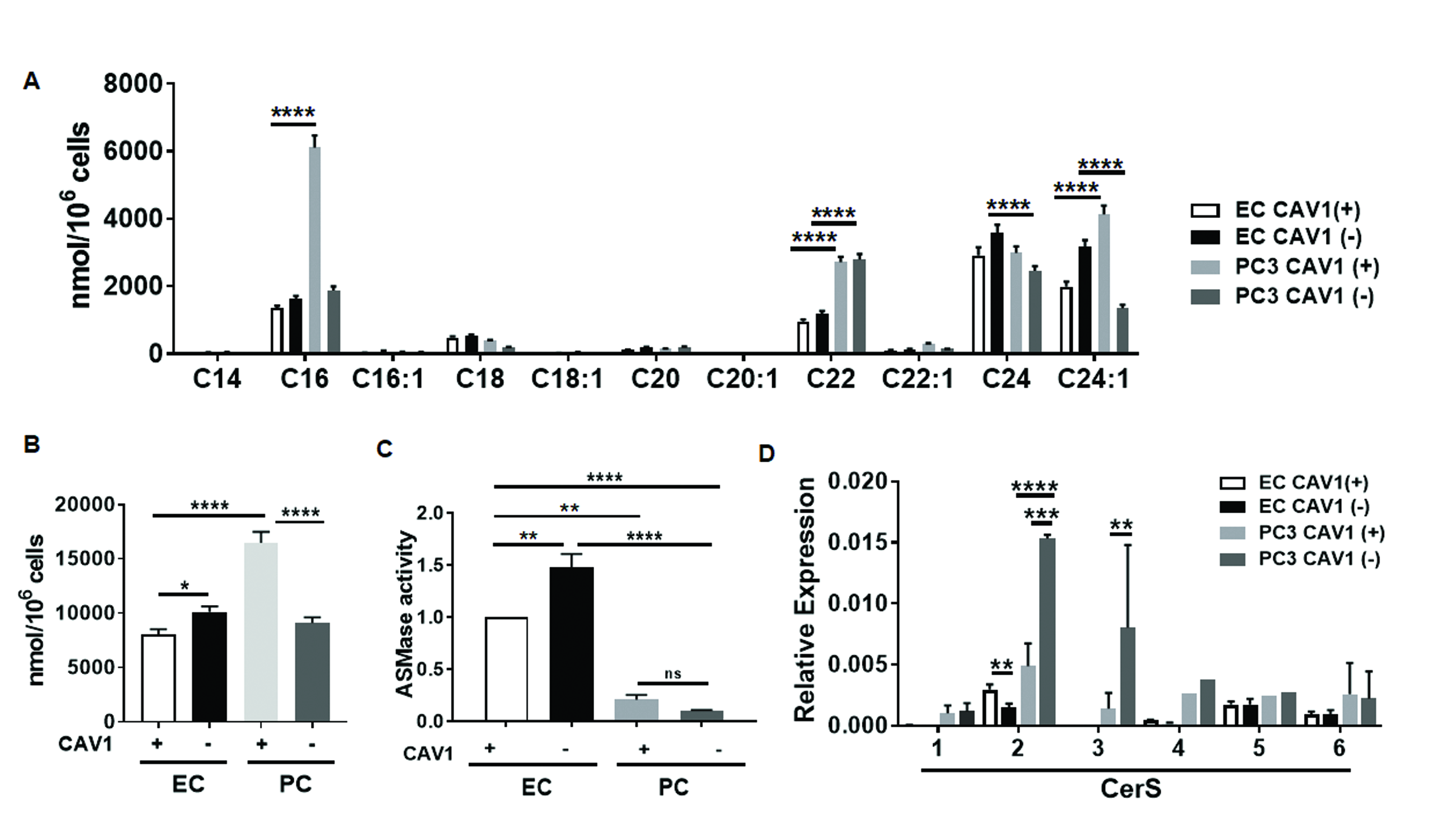

Supplement: Supplementary file 6 — Supplemental Figure S5 [file 41419_2020_2418_MOESM6_ESM.tif]

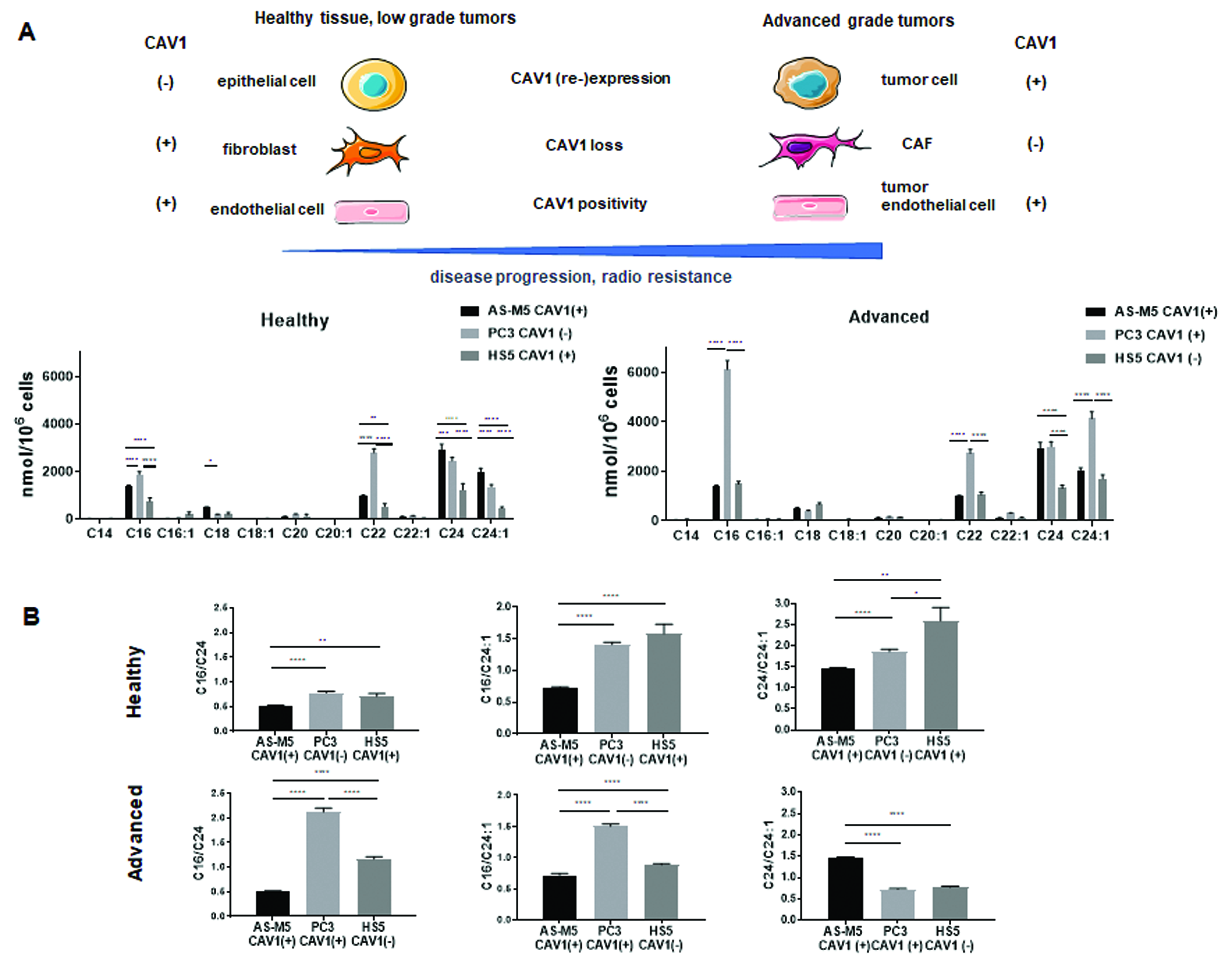

Supplement: Supplementary file 7 — Supplemental Figure S6 [file 41419_2020_2418_MOESM7_ESM.tif]
